# Antitumor Effect of Calcium-Mediated Destabilization of Epithelial Growth Factor Receptor on Non-Small Cell Lung Carcinoma

**DOI:** 10.3390/ijms19041158

**Published:** 2018-04-11

**Authors:** In Un Kim, In Sung Sung, Jae Jun Sim, Minhee Park, Keun-Yeong Jeong, Hwan Mook Kim

**Affiliations:** 1 Gachon Institute of Pharmaceutical Science, Gachon University, 191, Hambangmoe-ro, Yeonsu-gu, Incheon 21936, Korea; kiminun5@naver.com (I.U.K.); sltjd0114@naver.com (I.S.S.); genesis0804@hanmail.net (J.J.S.); 2 Metimedi Pharmaceuticals Co. R&D Division, 263, Central-ro, Yeonsu-Gu, Incheon 22006, Korea; pmh1880@hanmail.net

**Keywords:** non-small-cell lung carcinoma, epithelial growth factor receptor, Src, alpha-tubulin, calcium, calpain

## Abstract

Despite the development of numerous therapeutics targeting the epithelial growth factor receptor (EGFR) for non-small cell lung carcinoma (NSCLC), the application of these drugs is limited because of drug resistance. Here, we investigated the antitumor effect of calcium-mediated degradation of EGFR pathway-associated proteins on NSCLC. First, lactate calcium salt (LCS) was utilized for calcium supplementation. Src, α-tubulin and EGFR levels were measured after LSC treatment, and the proteins were visualized by immunocytochemistry. Calpeptin was used to confirm the calcium-mediated effect of LCS on NSCLC. Nuclear expression of c-Myc and cyclin D1 was determined to understand the underlying mechanism of signal inhibition following EGFR and Src destabilization. The colony formation assay and a xenograft animal model were used to confirm the in vitro and in vivo antitumor effects, respectively. LCS supplementation reduced Src and α-tubulin expression in NSCLC cells. EGFR was destabilized because of proteolysis of Src and α-tubulin. c-Myc and cyclin D1 expression levels were also reduced following the decrease in the transcriptional co-activation of EGFR and Src. Clonogenic ability and tumor growth were significantly inhibited by LSC treatment-induced EGFR destabilization. These results suggest that other than specifically targeting EGFR, proteolysis of associated molecules such as Src or α-tubulin may effectively exert an antitumor effect on NSCLC via EGFR destabilization. Therefore, LCS is expected to be a good candidate for developing novel anti-NSCLC therapeutics overcoming chemoresistance.

## 1. Introduction

In 2017, Korea witnessed 26,093 new cases of lung cancer, which is the most common cancer in males. Of these, 18,713 cases are projected to die, accounting for 27.4% of cancer-related deaths in males and 16.7% in females [1]. Approximately 80–85% cases of lung cancer were established as non-small cell lung carcinoma (NSCLC) [1,2].

The pathway of epithelial growth factor receptor (EGFR) signaling is important for regulating the growth, proliferation, invasion and metastasis of cancer cells, including NSCLC [3,4]. EGFR dimerizes upon activation by ligand, such as EGF, infrequently as heterodimers with other EGFR family members; it is also activated by phosphorylation and interacts with various intracellular proteins [5]. These proceedings activate signaling cascades in the cancer cells, and accordingly, the receptors are dimerized and internalized via endocytic trafficking and are moved to the cell surface for recycling or degraded by lysosomes [6,7]. Therefore, EGFR is an important molecular target for NSCLC therapy.

pp60c-Srs (Src) is a family of protein-tyrosine kinases involved in the control of signal transduction and trafficking of EGFR. Src has several noncatalytic domains that positively and negatively regulate Src tyrosine kinase activity. Phosphorylation of Src in the noncatalytic C-terminal tail is a key mechanism for the repression of Src tyrosine kinase activity [8]. Subsequent studies have also indicated Src to be crucial for cell proliferation, survival and migration. In this background, Src has been recognized to cooperate with EGFR and to be a significant determinant of EGFR-mediated oncogenesis [9]. Consistent with the results of these studies, EGFR and Src are often found to be co-overexpressed in human cancers, such as in NSCLC tissue [10]. Further, Src activity antagonizes the function of Cbl and inhibits EGFR proteolysis [11].

Microtubules, polymers of α- and β-tubulin, perform important functions for various cellular processes such as intracellular trafficking, cell movement, mitosis and cell division [12]. Microtubules also regulate EGFR endocytosis and stabilization by histone deacetylase 6-mediated control of acetylation and receptor trafficking along microtubules [13]. Disruption of the microtubule network is associated with EGFR dephosphorylation and subsequent reduced activation of cell signaling. Therefore, microtubules are ideal targets for anticancer drugs, and several reports mention the use of microtubule targeting drugs such as docetaxel and vincristine [14].

Lactate calcium salt (LCS) is a crystalline salt, which is formed by the complex of lactic acid on calcium carbonate. LCS is a chemical ingredient that has been used as a remedy for hypocalcemia treatment and listed as Generally Recognized As Safe by the U.S Food and Drug (FDA) Administration (GRAS) for use in food additives since the 1980s (Code of Federal Regulations Title 21 184.1207). This research has changed the use of this ingredient to investigate its anticancer efficacy [15]. Previously, we reported that sustained calcium influx by LCS causes a calpain-dependent increase in proteolysis of focal adhesion kinase, which is accompanied by reduction in the levels of proteins participating in the signaling cascade via focal adhesion kinase, including Src [16,17]. These results prompted the idea that LCS might exhibit antitumor effect via Src destabilization as Src regulates EGFR stabilization and downstream signaling in NSCLC cells [3,8,11].

In the present study, NSCLC cells were supplemented with LCS, and the antitumor effect of LCS, with a focus on calcium-mediated proteolysis of related factors associated with EGFR stabilization, was investigated.

## 2. Results

### 2.1. Src Is Destabilized in Non-Small Cell Lung Carcinoma (NSCLC) Cells after Lactate Calcium Salt (LCS) Treatment in a Time-Dependent Manner

**Figure 1:**
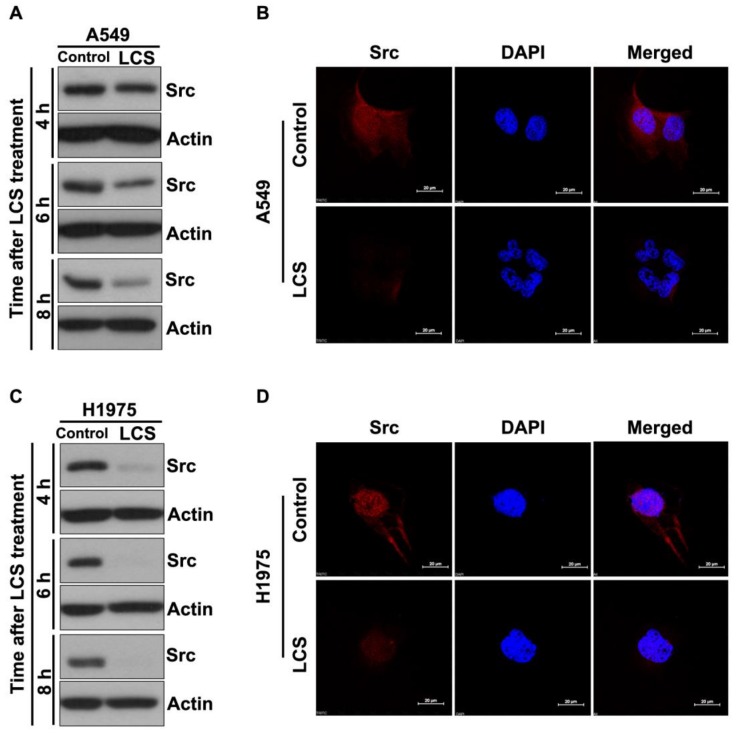
Lactate calcium salt (LCS)-mediated Src destabilization in non-small cell lung carcinoma cells. (**A**) Western blot analysis for Src expression in A549 cells following time-dependent LCS treatment. (**B**) Immunocytochemical analysis for Src expression in A549 cells following LCS treatment. (**C**) Western blot analysis for Src expression in H1975 cells following time-dependent LCS treatment. (**D**) Immunocytochemical analysis for Src expression in H1975 cells following LCS treatment. Scale bar: 20 µm. DAPI, 4′,6-diamidino-2-phenylindole.

Src has a critical role in the altered endocytic trafficking of EGFR via the recycling pathway and enables prolonged signaling via EGFR [18]. To investigate the effect of LCS on Src stabilization, Src expression was determined in NSCLC cells after treatment with 2.5 mM LCS for 4, 6 and 8 h under hypoxic condition (Figure 1A,C). Western blotting showed that Src expression gradually decreased in A549 (Figure 1A) and H1975 (Figure 1C) cells after LCS treatment in a time-dependent manner (Figure 1A,C). Src expression was visualized by immunocytochemistry (ICC) after treatment with 2.5 mM LCS for 8 h to evaluate Src stabilization in NSCLC cells (Figure 1B,D). Results show that the LCS treatment reduced Src expression in A549 (Figure 1B) and H1975 (Figure 1D) cells.

### 2.2. LCS Treatment Destabilizes α-Tubulin in NSCLC Cells in a Time-Dependent Manner

**Figure 2:**
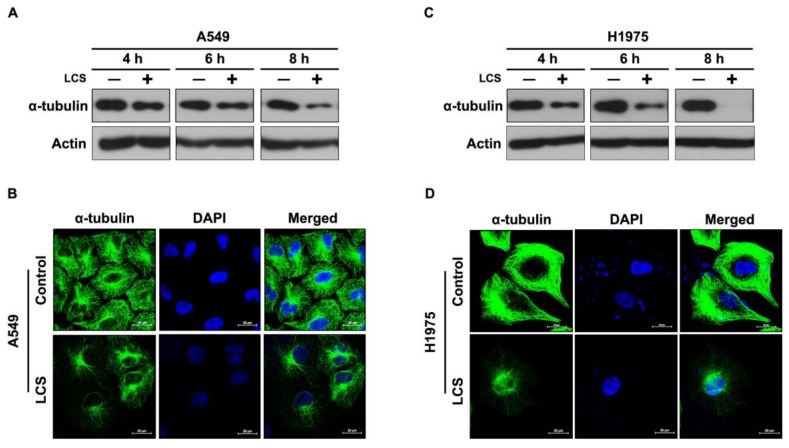
Lactate calcium salt (LCS)-mediated α-tubulin degradation in non-small cell lung carcinoma cells under hypoxia. (**A**) Western blot analysis for α-tubulin expression in A549 cells following time-dependent LCS treatment. (**B**) Immunocytochemical analysis for α-tubulin expression in A549 cells following LCS treatment. (**C**) Western blot analysis for α-tubulin expression in H1975 cells following time-dependent LCS treatment. (**D**) Immunocytochemical analysis for α-tubulin expression in H1975 cells following LCS treatment. Scale bar: 20 µm.

Microtubules form a functional network for intracellular signaling and/or trafficking of EGFR [19]. To investigate the effects of LCS on α-tubulin stabilization, α-tubulin expression was evaluated in NSCLC cells after treatment with 2.5 mM LCS for 4, 6 and 8 h under hypoxic condition (Figure 2A,C). Western blotting showed that α-tubulin expression gradually decreased in A549 (Figure 2A) and H1975 (Figure 2C) cells following LCS treatment in a time-dependent manner (Figure 2A,C). To evaluate α-tubulin stability in NSCLC cells, α-tubulin expression was visualized by ICC after treatment with 2.5 mM LCS for 8 h (Figure 2B,D). Results show that LCS treatment reduced α-tubulin expression in A549 (Figure 2B) and H1975 (Figure 2D) cells.

### 2.3. EGFR Is Destabilized in NSCLC Cells Following LCS-Mediated Src and α-Tubulin Degradation

**Figure 3:**
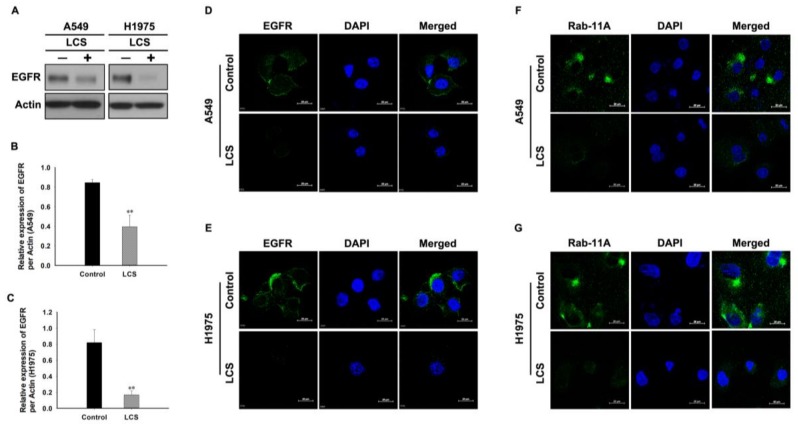
Epithelial growth factor receptor (EGFR) and Rab-11A degradation in non-small cell lung carcinoma cells by lactate calcium salt (LCS) treatment. (**A**) Western blot analysis for EGFR expression in A549 and H1975 cells following LCS treatment. (**B**) Quantitative analysis of EGFR expression in A549 following LCS treatment. The results shown are averages of triplicate analyses. (**C**) Quantitative analysis of EGFR expression in H1975 cells following LCS treatment under hypoxia. The results shown are the averages of triplicate analyses. (**D**) Immunocytochemical analysis for EGFR expression in A549 cells following LCS treatment. (**E**) Immunocytochemical analysis for EGFR expression in H1975 cells following LCS treatment. (**F**) Immunocytochemical analysis for Rab-11A expression in A549 cells following LCS treatment. (**G**) Immunocytochemical analysis for Rab-11A expression in H1975 cells following LCS treatment. Results are the mean ± SD. ** *p* < 0.001 vs. control. Scale bar: 20 µm.

To investigate the effects of LCS on EGFR stability, EGFR expression was determined in NSCLC cells after treatment with 2.5 mM LCS for 8 h under hypoxic condition (Figure 3). Western blotting analysis showed that LCS treatment reduced EGFR expression in A549 and H1975 cells (Figure 3A). Quantitative analysis of the relative expression of EGFR in A549 and H1975 cells indicated that LCS treatment significantly reduced the expression ratio of EGFR with respect to actin (Figure 3B,C). The ratio decreased to 0.5 ± 0.12 and 0.2 ± 0.15 in A549 and H1975 cells, respectively (Figure 3B,C). EGFR and Rab-11A expression was visualized by ICC after treatment with 2.5 mM LCS for 8 h to evaluate EGFR destabilization in NSCLC cells (Figure 3D,E). Rab-11A, a significant molecule for vesicular trafficking, plays an important role in invasion signaling and membrane protein recycling in cancer cells [20]. Results show that LCS treatment reduced EGFR and Rab-11A expression in A549 (Figure 3D,F) and H1975 (Figure 3E,G) cells.

### 2.4. Destabilization of Src and EGFR by LCS Is Mediated by Calcium-Dependent Calpain Activation

**Figure 4:**
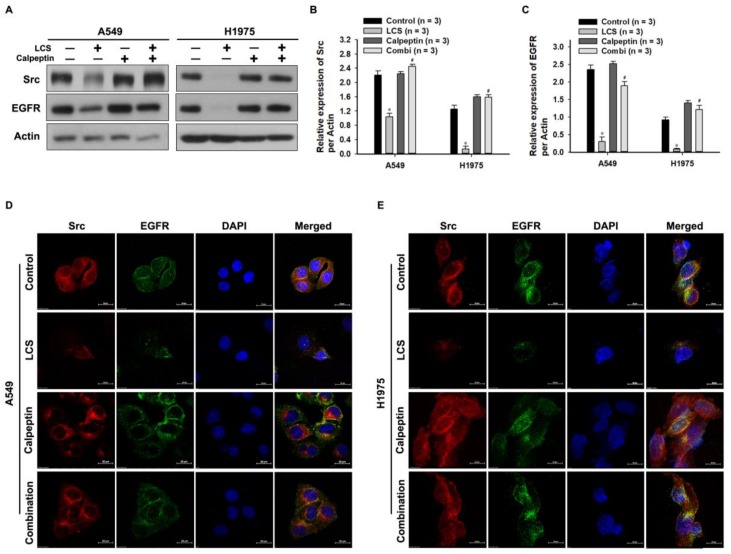
Calcium-mediated destabilization of Src and epithelial growth factor receptor (EGFR) in non-small cell lung carcinoma (NSCLC) cells. (**A**) Western blot analysis for evaluating the effect of calcium-mediated calpain activity on Src and EGFR expression in NSCLC cells under normoxia. (**B**,**C**) Quantitative analysis for Src and EGFR expression in NSCLC cells. Results are the mean ± SD. * *p* < 0.001 vs. control; # *p* < 0.001 vs. LCS. (**D**,**E**) Immunocytochemical analysis for determining the effect of calcium-mediated calpain activity on Src and EGFR expression in NSCLC cells. Calpeptin is a protein belonging to the family of calcium-dependent proteolytic enzymes, which was used for confirming the calcium-mediated effect of LCS on NSCLC.

To investigate the effect of calcium on Src destabilization, NSCLC cells were co-treated with 2.5 mM LCS and calpeptin, a potent inhibitor of calpain activity, under hypoxic condition (Figure 4), and subsequently, the Src and EGFR levels were quantitatively analyzed (Figure 4A–C). Western blotting indicated that LCS treatment reduced Src and EGFR levels; however, the calcium-mediated effect on calpain activity was reduced by co-treatment with calpeptin and LCS (Figure 4A). Compared to the control, calpeptin treatment alone did not change Src and EGFR levels (Figure 4A). Quantitative analysis indicated that the expression ratios of Src and EGFR with respect to actin were significantly reduced by LCS treatment (Figure 4B,C). The expression ratio of Src decreased from 2.21 ± 0.12 to 1.03 ± 0.10 and from 1.25 ± 0.11 to 0.13 ± 0.08 in A549 and H1975 cells, respectively (Figure 4B). After co-treatment with calpeptin and LCS, the expression ratio recovered to 2.45 ± 0.06 and to 1.58 ± 0.07 in A549 and H1975 cells, respectively (Figure 4B). The expression ratio of EGFR decreased from 2.35 ± 0.13 to 0.30 ± 0.12 and from 0.92 ± 0.08 to 0.08 ± 0.025 in A549 and H1975 cells, respectively (Figure 4C). After co-treatment with calpeptin and LCS, the expression ratio of EGFR recovered to 1.90 ± 0.11 and 1.21 ± 0.12 in A549 and H1975 cells, respectively (Figure 4C). ICC was used to visualize Src and EGFR co-expression and to evaluate their stability (Figure 4D,E). LCS treatment simultaneously reduced Src and EGFR levels; however, the calcium-mediated effect was reduced in the group co-treated with calpeptin and LCS (Figure 4D,E).

### 2.5. Destabilization of α-Tubulin by LCS Is Mediated by Calcium-Dependent Calpain Activation

**Figure 5:**
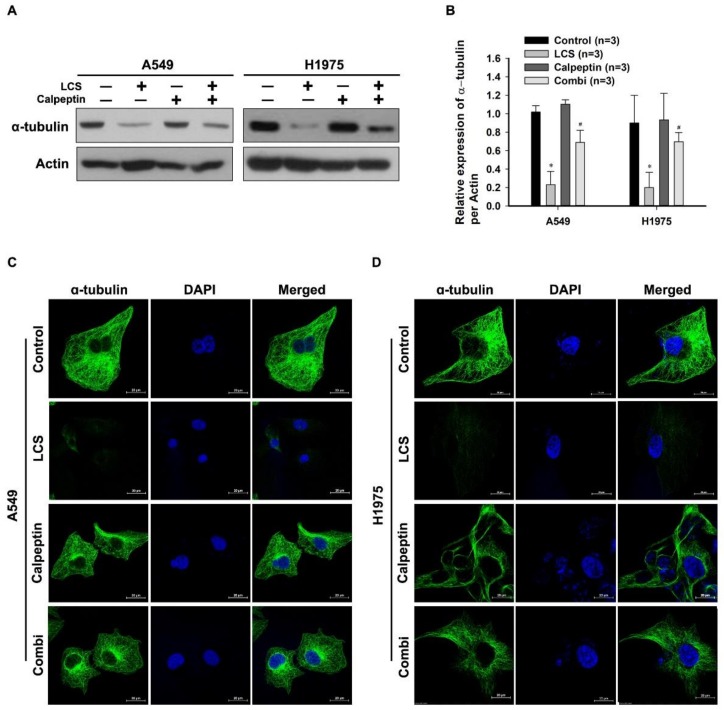
Calcium-mediated α-tubulin degradation in non-small cell lung carcinoma cells. (**A**) Western blot analysis for determining the effect of calcium-mediated calpain activity on α-tubulin expression in NSCLC cells. (**B**) Quantitative analysis for α-tubulin levels in NSCLC cells. Results are the mean ± SD. * p < 0.001 vs. control; *# p* < 0.001 vs. LCS. (**C**,**D**) Immunocytochemical analysis for evaluating the effect of calcium-mediated calpain activity on α-tubulin expression in NSCLC cells. Calpeptin is a protein belonging to the family of calcium-dependent proteolytic enzymes, which was used for confirming the calcium-mediated effect of LCS on NSCLC.

Calpain can also hydrolyze cytoskeletal structures such as microtubules [14]. To investigate the effect of calcium on α-tubulin destabilization, NSCLC cells were co-treated with 2.5 mM LCS and calpeptin under hypoxia, followed by quantitative analysis of α-tubulin levels (Figure 5A,B). Western blotting indicated that LCS treatment reduced α-tubulin expression; however, the calcium-mediated effect was reduced by co-treatment with calpeptin and LCS (Figure 5A). Compared to the control, calpeptin treatment alone did not change α-tubulin expression (Figure 5A). Quantitative analysis indicated that LCS treatment significantly reduced the expression ratio of α-tubulin to actin (Figure 5B). The expression ratio of α-tubulin decreased to 0.23 ± 0.14 and 0.2 ± 0.16 in A549 and H1975 cells, respectively (Figure 5B). After co-treatment with calpeptin and LCS, the expression ratio recovered to 0.68 ± 0.13 and 0.69 ± 0.1 in A549 and H1975 cells, respectively (Figure 5B). The effect of LCS on α-tubulin expression in NSCLC was visualized by ICC (Figure 5C,D). α-tubulin expression was decreased by LCS treatment; however, the calcium-mediated effect of LCS was reduced by combined treatment with calpeptin and LCS (Figure 5C,D).

### 2.6. Nuclear Signaling in NSCLC Is Reduced Following Calcium-Mediated Destabilization of EGFR and Src

**Figure 6:**
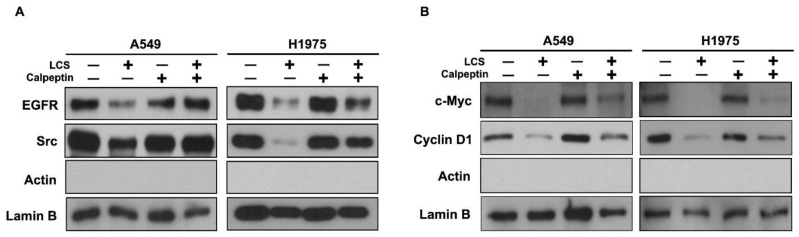
Nuclear signaling in non-small cell lung carcinoma (NSCLC) cells. (**A**) Western blot analysis for nuclear expression of the epithelial growth factor receptor (EGFR) and Src in NSCLC cells. (**B**) Western blot analysis for nuclear expression of c-Myc and cyclin D1 in NSCLC cells. Calpeptin is a protein belonging to the family of calcium-dependent proteolytic enzymes that was used for confirming the calcium-mediated effect of LCS on NSCLC. Lamin B was used as a loading control for the nuclear extracts.

EGFR and Src act as transcription co-activators for numerous oncogenic genes, including those encoding c-Myc and cyclin D1 [19]. To investigate the inhibition of signal transduction in the nucleus after EGFR and Src destabilization, the signaling pathway was evaluated in the nuclear extracts of NSCLC cells (Figure 6). Nuclear EGFR and Src were destabilized by LCS treatment; however, the effect was reduced after co-treatment with calpeptin and LCS, whereas calpeptin alone did not elicit any change (Figure 6A). c-Myc and cyclin D1 levels decreased in the nuclear extracts after EGFR and Src destabilization. However, calpeptin led to the recovery of nuclear signaling (Figure 6B).

### 2.7. LCS Treatment Reduces Proliferation of NSCLC Cells

**Figure 7:**
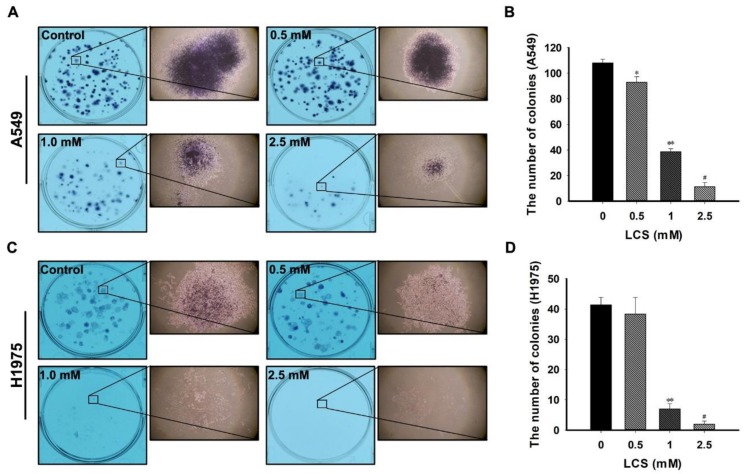
Clonogenic ability of non-small cell lung carcinoma (NSCLC) cells to confirm the inhibitory effect of lactate calcium salt (LCS) on cell proliferation. (**A**) Representative images of A549 colonies following treatment with 0.5, 1 and 2.5 mM LCS. (**B**) Quantitative analysis for the number of A549 colonies. Results are the averages of triplicate analyses. (**C**) Representative images of H1975 colonies following treatment with 0.5, 1 and 2.5 mM LCS. (**D**) Quantitative analysis for the number of H1975 colonies. Results are the averages of triplicate analyses. Results are the mean ± SD. * *p* < 0.05 vs. control (0 mM); ** *p* < 0.001 vs. 0 and 0.5 mM LCS; # *p* < 0.001 vs. 0, 0.5, and 1 mM LCS.

To investigate the anti-tumor effect of LCS on NSCLC cells, clonogenic ability was observed after treatment with different doses of LCS. The number of colonies was counted to determine clonogenicity (Figure 7). Figure 7A,C represents the NSCLC colonies obtained following treatment with 0.5, 1 and 2.5 mM LCS. A single colony was magnified to investigate the morphological features (Figure 7A,C). The shape of the single colony was incompact and smaller than that of the control after treatment with 2.5 mM LCS (Figure 7A,C). The number of colonies was significantly reduced in a dose-dependent manner (Figure 7B,D) to 11.3 ± 3.2 and to 2.0 ± 1.0 in A549 and H1975 cells, respectively, whereas those in the control group were 108.0 ± 3.0 (A549) and 41.3 ± 2.5 (H1975) (Figure 7B,D).

### 2.8. The In Vivo Antitumor Effect of LCS on NSCLC Is Induced by EGFR and Src Destabilization

**Figure 8:**
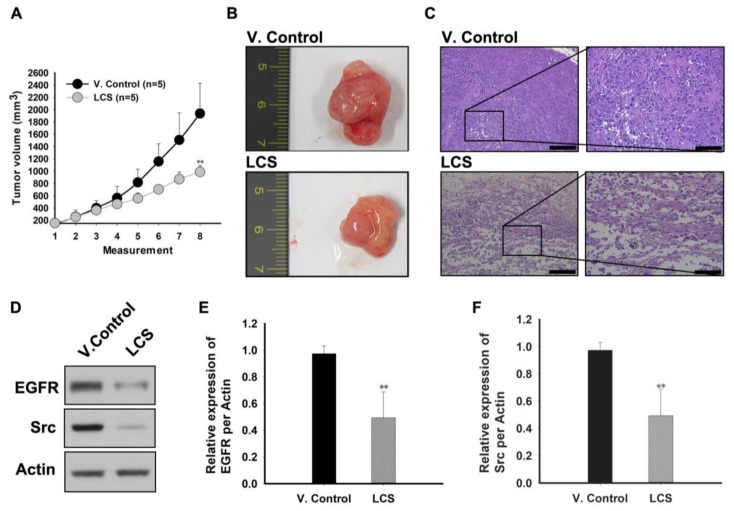

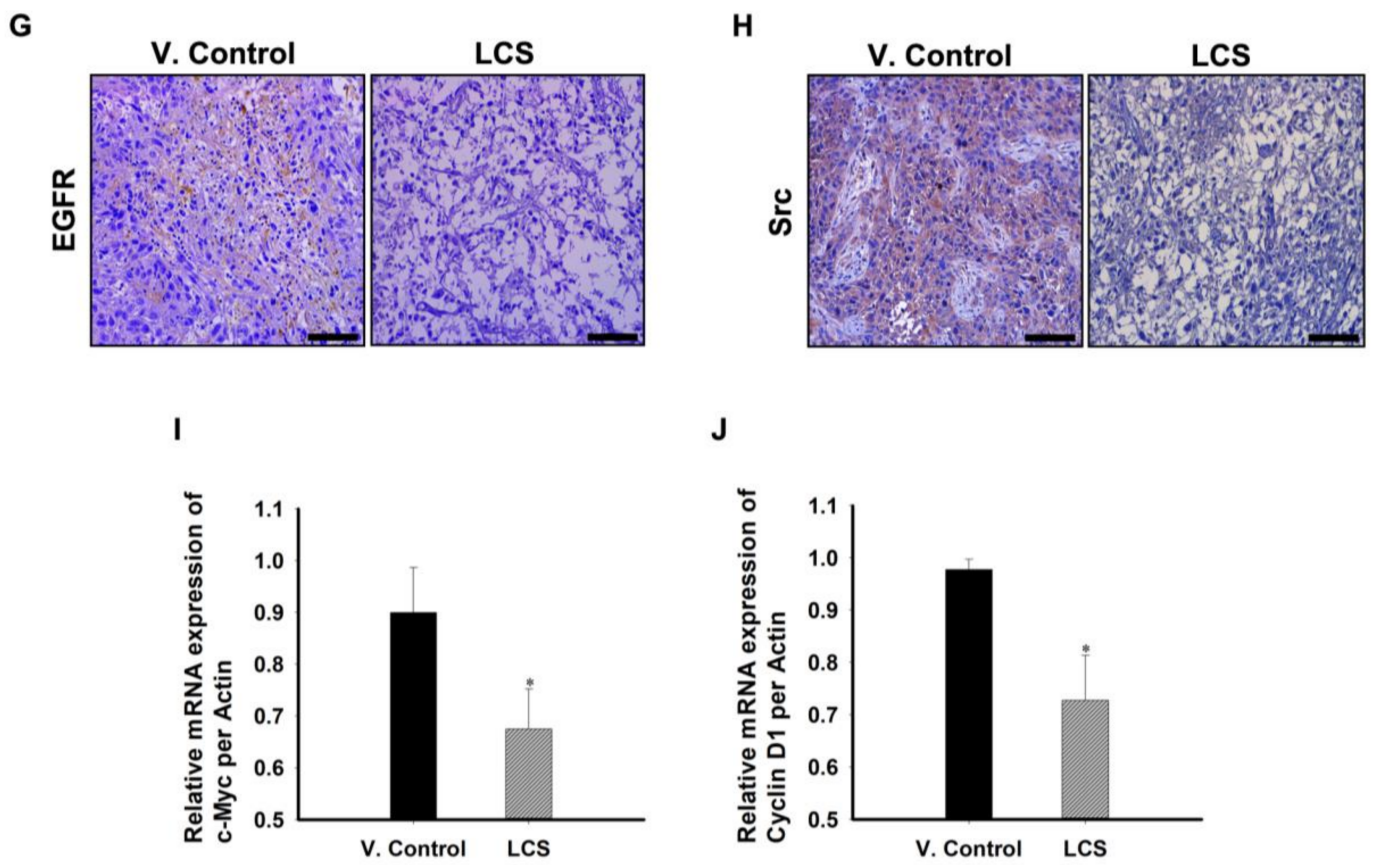
Lactate calcium salt (LCS)-mediated in vivo antitumor effect on non-small cell lung carcinoma. (**A**) Comparison of tumor growth in the heterotopic xenograft mice. (**B**) Representative images of tumor mass between the vehicle control (V. control) and LCS-treated groups. (**C**) Hematoxylin and eosin (H&E) analysis for comparison of tumor necrosis between V. control and LCS treatment. Left scale bars: 100 µm; Right scale bars 50 µm. (**D**) Western blot analysis for the epithelial growth factor receptor (EGFR) and Src expression in the tumor tissue. (**E**) Quantitative analysis for comparison of EGFR expression in the tumor tissue between the control and LCS-treated group. (**F**) Quantitative analysis for comparison of Src expression in the tumor tissue between the control and LCS treatment group. (**G**,**H**) Immunohistochemical analysis for comparison of EGFR and Src expression between the control and LCS-treated group. Scale bars: 50 µm. (**I**,**J**) Quantitative analysis for comparison of the mRNA levels of c-Myc and cyclin D1 in the tumor tissue between the control and LCS-treated group. The results on each graph represent values analyzed for three samples. * *p* < 0.05 vs. control; ** p < 0.001 vs. control. Scale bar: 100 µm. Results are the mean ± SD.

To confirm the in vivo antitumor effect of LCS, tumor volumes were chronologically measured (three times per week) using a heterotopic xenograft animal model (Figure 8A–C). In the LCS-treated group, tumor volume significantly decreased from 12 days after the measurement (fifth measurement on Figure 8A) compared to that in the vehicle control (V. control) group (Figure 8A). On the last day of measurement, tumor volume was 981.18 ± 103.27 mm^3^ (average) in the LCS-treated group, whereas it was 1987.491 ± 491.77 mm^3^ (average) in the V. control group (Figure 8A). The representative images comparing tumor growth between the V. control and LCS-treated group are show in Figure 8B. The internal structure of the LCS-treated tumor was collapsed, and nuclear staining was largely undetected. Following LCS treatment, necrosis was induced in the whole tumor mass, including periphery of the tumor (Figure 8C).

To elucidate the mechanism underlying the in vivo antitumor effect of LCS, we determined the levels of proteins that were related to the in vitro antitumor mechanism in the tumor tissue (Figure 8D–J). EGFR and Src levels were reduced in the LCS-treated group (Figure 8D). Quantitative analysis of the relative expression of the EGFR and Src indicated that the expression ratio with respect to actin was significantly decreased to 0.5 ± 0.19 and to 0.3 ± 0.27 in the LCS-treated group, respectively (Figure 8E,F). EGFR and Src expression were visualized by immunohistochemical staining to determine EGFR and Src stability in the tumor tissue (Figure 8G,H). The results show that EGFR and Src expression in the tumor tissue was reduced in the LCS-treated group (Figure 8G,H). Finally, to investigate EGFR and Src transcription in the tumor, mRNA extracted from the tumor tissue was used to determine the expression of the c-Myc and cyclin D1 by quantitative reverse transcription-polymerase chain reaction (qRT-PCR) (Figure 8I,J). Quantitative analysis of the relative expression of c-Myc and cyclin D1 indicated that the expression ratio of c-Myc and cyclin D1 with respect to actin was significantly decreased to 0.68 ± 0.08 and 0.73 ± 0.09 in the LCS-treated group, respectively (Figure 8I,J).

## 3. Discussion

In this study, we observed that LCS showed potent antitumor activity in NSCLC. Calcium-mediated calpain activation induced EGFR destabilization following Src and α-tubulin degradation. A decrease in the level of Rab-11A, a tumor malignancy and EGFR recycling marker of the NSCLC, was also identified.

First, we investigated the mechanism underlying the antitumor effect of LCS. Since calcium is the main component of LCS, we determined the effect of calcium on NSCLC cells. Calpain is an evolutionarily-conserved cysteine protease activated by high levels of intracellular calcium [21]. Our previous results indicated that calcium influx by LCS causes calpain-mediated FAK and Src proteolysis in cancer cells, which is supplemented by a reduction in the levels of proteins participating in the protein kinase activity [15,16,17]. These observations prompted the idea that LCS might exert an antitumor effect on NSCLC via Src inhibition because Src is a known regulator of EGFR activation and recycling in NSCLC cells [18].

NSCLC patients with EGFR mutations inescapably develop acquired resistance following treatment with EGFR tyrosine kinase inhibitors (TKIs). Although recent advances in chemotherapy and targeted therapy have offered new treatment options for TKI-resistant NSCLC, the development stage is early with unclear effects [22]. Therefore, anticancer drugs that can overcome TKI resistance and generally target NSCLC are required. Calcium-dependent calpain activation can affect NSCLC irrespective of the existence of EGFR mutations. Therefore, LCS can be used as an anticancer drug regardless of the NSCLC cell type, such as on A549 (TKI-sensitive) and H1975 (TKI-resistant) cells.

Src is a cytosolic non-receptor tyrosine kinase that transduces signals between the cell surface and other intracellular proteins [23]. Dual expression of Src and EGFR occurs in most cancer cells, which has been correlated with Src-dependent increase in the activation of EGFR downstream signaling [18,24]. Src phosphorylates tyrosine 845 (Y845) on EGFR, which induces proliferation, survival and metabolism required for cancer progression [25]. Further, Src activity antagonizes the proteolysis of EGFR via Cbl activation [11]. As described previously [15,16,17], LCS treatment reduced Src expression in NSCLC.

Microtubules are dynamic structures involved in various cellular functions, such as intracellular trafficking of the EGFR [13]. Endocytic processing and receptor recycling involve microtubule-dependent vesicle transport [13,26]. α-tubulin levels regulate EGFR endocytosis and degradation and, consequently, receptor trafficking along microtubules [27]. Therefore, destabilization of α-tubulin by LCS treatment was an important determinant of the antitumor effect on NSCLC.

Calpains are intracellular cysteine proteases that are activated by calcium influx [28]. Therefore, calpeptin, a calpain inhibitor, was used to determine whether calcium-mediated calpain activity was directly induced by LCS. Our results show that calpeptin treatment reduced the anticancer efficacy of LCS and restored the expression of Src, α-tubulin and EGFR. These results corroborated the observation that LCS-induced calcium-mediated calpain activity plays a decisive role in determining the anticancer efficacy of LCS in NSCLC. Although activation of the calcium-dependent calpain is antagonistic to the surrounding molecules relating to EGFR activation that we present through this study, further study for proving the broad action of calpain is needed. Because calpain is a protease that can mediate the degradation of several intracellular proteins, it has been subjected to proving whether the proposed concentration and mechanism result in toxicity to normal cells [29]. Additionally, further clarification of the mechanism of Src and α-tubulin degradation through LCS-mediated calpain activation is also needed.

Rab-11a is a vesicle trafficking protein, which plays essential roles in various aspects of membrane trafficking control [20]. Receptor recycling, such as EGFR, can occur either via the Rab family-regulated route to the cancer cell membrane or specifically via a Rab11-dependent route to the endocytic recycling compartment [20,30]. The Rab11 supports indirect recycling from the early endosome to the perinuclear endocytic recycling compartment through the trans-Golgi network during the recycling process [30]. The molecular mechanism of Rab-11A function remains incompletely defined; however, it is well known that Rab coupling proteins are selectively degraded by calpain [30,31]. Therefore, reduction in Rab-11A expression by LCS treatment inhibited NSCLC cell malignancy [20].

Nuclear EGFR and Src play important roles in transcriptional co-activation of several cancer-promoting genes such as c-Myc and cyclin D1 [19]. Cyclin D1 is a cell cycle regulator; therefore, overexpression of cyclin D1 shortens G1 and accelerates cell proliferation [32]. c-Myc is located in many signal transduction pathways and is an immediate early response gene that acts as a signaling cascade of various complexes of ligand-membrane receptors [33]. Therefore, reduction in the expression of c-Myc and cyclin D1 is important for the inhibition of signaling involved in NSCLC progression [34,35].

Since the development of anticancer drugs targeting TKI-resistant NSCLC is a major clinical issue, TKI-resistant NSCLC cells were used to generate a xenograft animal model that was used to investigate the antitumor effect of LCS [36]. In this study, 2 mg/kg/day of LCS were used for in vivo administration. This was a very low dose, as the human equivalent dose is 10 mg/person/day; therefore there was no toxicity in the animal study. Currently, LCS is used in a treatment for hypocalcemia as a maximum up to 1200 mg/person/day. Our results show that tumor volume was significantly inhibited by daily treatment with LCS, which corroborated the observed calcium-mediated in vitro antitumor mechanism. In addition, we confirmed that expression levels of EGFR and Src and those of their target genes encoding c-Myc and cyclin D1 were also significantly inhibited in the tumor. In conclusion, most anticancer drugs for NSCLC treatment are developed as targeting agents, such as TKIs. However, these drugs can induce drug resistance after continuous treatment [22,36], and the majority of patients show disease progression within one to two years after treatment initiation because of TKI resistance. In addition to the specific targeting of EGFR, proteolysis of EGFR-associated molecules such as Src or α-tubulin may effectively exert an antitumor effect via EGFR destabilization. We demonstrated that calpain activation by intercellular calcium influx might be an important strategy for treating NSCLC and that LCS may be a good candidate for developing novel therapeutics.

## 4. Materials and Methods

### 4.1. Cell Lines and Culture Conditions

Human NSCLC cell lines (A549 and H1975) were purchased from the American Type Culture Collection (Manassas, VA, USA). The cells were maintained in Roswell Park Memorial Institute-1640 medium (Hyclone Laboratories, Logan, UT, USA) supplemented with 10% fetal bovine serum (Hyclone Laboratories), 100 IU/mL penicillin, and 100 µg/ mL streptomycin (Welgene, Daegu, Korea) in a humidified atmosphere of 5% CO_2_ at 37 °C. All in vitro experiments were performed under two conditions: one was a normal condition as described above and the other was a hypoxic condition that was maintained at 1% O_2_, 5% CO_2_ and 94% nitrogen.

### 4.2. Reagents

LCS and calpeptin were purchased from Sigma-Aldrich (St. Louis, MO, USA). LCS was dissolved in distilled water. Aqueous LCS was stored at 4 °C before treatment. Calpeptin was dissolved in dimethyl sulfoxide. Aqueous calpeptin was stored at 4 °C before treatment.

### 4.3. Protein Extraction

A549 and H1975 cells (3.0 × 10^5^ cells/well) were seeded in a 6-well plate. One day after incubation, the cells were treated with 2.5 mM LCS for 4, 6 and 8 h. Then, the cells were washed once with cold phosphate-buffered saline (PBS). To prepare whole cell lysate, cells were lysed by incubation in lysis buffer (1% NP-40, 0.25% sodium deoxycholate, 150 mM NaCl, 1 mM EDTA, 1% Triton X-100, 50 mM Tris-HCl (pH 7.4), 10% glycerol) containing a protease inhibitor cocktail (Roche, Basel, Switzerland) and phosphatase inhibitors (Na_3_VO_4_ 1 mM, NaF 100 mM, NaPP 10 mM) for 30 min at 4 °C, followed by centrifugation for 20 min at 10,000× *g* and 4 °C. The supernatant was used for Western blot analysis. Nuclear protein was prepared using a nuclear extraction kit (Abcam, Cambridge, MA, USA) according to the manufacturer’s instructions.

### 4.4. Western Blot Analysis

Quantitative analysis for the extracted protein and nuclear lysate was performed using the bicinchoninic acid assay kit (Thermo Scientific, Waltham, MA, USA). The proteins were boiled for 5 min at 95 °C and separated on 10 or 12% sodium dodecyl sulfate polyacrylamide gel, followed by transfer to polyvinylidene fluoride membranes (Merck Millipore, Billerica, MA, USA). After blocking in 5% non-fat milk (Bio-Rad, Hercules, CA, USA) for 2 h, the membranes were incubated overnight at 4 °C with primary antibody diluted in Tris-buffered saline containing Tween 20 (TBST), 5% bovine serum albumin (BSA) and 0.1% sodium azide (Sigma-Aldrich, St. Louis, MO, USA). Specific primary antibodies were used as follows: EGFR (1:1,000, Cell Signaling, Danvers, MA, USA); Src (1:1000, Cell Signaling); actin (1:1000, Santa Cruz Biotechnology, Santa Cruz, CA, USA); α-tubulin (1:10,000, Merck Millipore, Billerica, MA, USA); c-Myc (1:1000, Cell Signaling Biotechnology); cyclin D1 (1:1000, Cell Signaling Biotechnology); lamin B (1:1000, Santa Cruz Biotechnology). The membranes were washed with TBST and incubated with secondary antibodies for 2 h. The secondary antibodies used were as follows: anti-rabbit secondary antibody (1:10,000, Abclone, Seoul, Korea); anti-mouse secondary antibody (1:10,000, Abclone); anti-goat secondary antibody (1:5000, Abclone). Immunoblots were developed using a detection reagent (Abclone) and exposed to an X-ray film (Agfa, Leverkusen, Germany) according to the manufacturer’s protocol. The relative amounts as a ratio of each protein band relative to the lane’s loading control were quantified by the ImageJ program [37].

### 4.5. Immunocytochemistry

A549 and H1975 cells were cultured on a bio-coated cover slip (BD Bioscience, San Jose, CA, USA) and fixed using 4% paraformaldehyde for 20 min. Subsequently, the cover slips were incubated with the primary antibodies for 15 h at 4 °C. The primary antibodies used were as follows: EGFR (1:300, Santa Cruz Biotechnology); Src (1:300, Cell Signaling Technology); α-tubulin (1:300, Merck Millipore); Rab-11A (1:300, Santa Cruz Biotechnology). After a PBS wash, the cells were incubated with anti-rabbit secondary biotinylated antibody (1:2000, Vector Laboratories, Burlingame, CA, USA) and visualized using streptavidin conjugated to fluorescein (Vector Laboratories, Burlingame, CA, USA). For double immunostaining, the cells were incubated with anti-mouse secondary biotinylated antibody (1:2000, Vector Laboratories, Burlingame, CA, USA) and visualized using streptavidin conjugated to fluorescein (Vector Laboratories, Burlingame, CA, USA). The coverslips were mounted on microscope slides with VECTASHIELD^®^ Hard Set™ mounting medium with 4′,6-diamidino-2-phenylindole (DAPI) (Vector Laboratories, Burlingame, CA, USA). Confocal fluorescence images were obtained by confocal laser scanning microscopy (LSCM, Nikon A1+, Tokyo, Japan) using an oil immersion lens.

### 4.6. Colony Formation Assay

A549 and H1975 cells (5 × 10^2^ cells/well) were seeded in a 6-well plate. After 24 h, the cells were treated with 0.5, 1 and 2.5 mM LCS and incubated for 14 days. The colonies were fixed using 100% methanol and stained by hematoxylin (Thermo Fisher Scientific, Waltham, MA, USA). The colonies were counted under an optical microscope (Olympus, Center Valley, PA, USA).

### 4.7. Animals

All experiments were performed under the institutional guidelines established by the Institutional Animal Care and Use Committee of the Gachon University (IACUC-2016-0053, 22 June 2016). Five-week-old Balb/c female nude mice were purchased from Charles River Laboratories (Wilmington, MA, USA). All animals were maintained in a 12-h light/dark cycle (light on, 08:00 h) at 22 to 25 °C, with free access to food and water.

### 4.8. Xenograft Animal Model

H1975 cells (1 × 10^7^) were inoculated into the hind flank of mice, and tumor size was measured three times per week. Tumor size was determined using digital calipers, and the tumor volume was calculated using the following formula: V = (L × W^2^)/2 (L, length; W, width). The mice were treated subcutaneously with 2 mg/kg/day LCS for 21 days daily when the tumor grew to 150 to 200 mm^3^. At the end of the experiment, all tumors were harvested and used for molecular biological and histological analysis.

### 4.9. Immunohistochemistry

The tumor tissues were fixed with 10% neutral buffered formalin and embedded in paraffin for sectioning. Tissue sections (5 μm thick) were cut from the paraffin-embedded blocks using a microtome. The sectioned tissues were incubated at 60 °C for 2 h and subsequently deparaffinized in xylene and dehydrated in a graded ethanol series. Endogenous peroxidase activity was blocked with 3% hydrogen peroxide in distilled water for 30 min. Heat-induced antigen retrieval was performed in 10 mM citrate buffer (pH 6.0) in a microwave oven, followed by washing thrice with PBS. The sections were blocked using a blocking agent (Invitrogen, Frederick, MD, USA) and incubated overnight with the primary antibodies at 4 °C. The primary antibodies used were as follows: EGFR (1:100, Santa Cruz Biotechnology) and Src (1:100, Santa Cruz Biotechnology). The sections were washed with PBST and incubated with biotinylated anti-mouse antibody (1:500, Vector Laboratories, Burlingame, CA, USA) for 1 h. After a PBST wash, the sections were treated with 3,3′-diaminobenzidine substrate (Dako, Carpentaria, CA, USA) and counterstained with hematoxylin (Thermo Fisher Scientific, Waltham, MA, USA). A Leica DM 1000 LED microscope (Leica Microsystems, Wetzlar, Germany) was used for image analysis.

### 4.10. Quantitative Reverse Transcription-Polymerase Chain Reaction

TRIzol reagent (Invitrogen, Carlsbad, CA, USA) was used for the extraction of total RNA from the tumor tissues according to the manufacturer’s protocol. Next, cDNA was synthesized from 2 μg total RNA using the PrimeScript^®^ first strand cDNA synthesis kit (Takara, Kusatsu, Japan). Actin was used as a constitutive control for normalization. cDNA was amplified using specific primers. qRT-PCR was performed using the SYBR premix ExTaq system (Takara, Kusatsu, Japan) and Stratagene Mx3005P detector (Agilent Technologies, Santa Clara, CA, USA). PCR conditions included one cycle of 10 min at 95 °C followed by 40 cycles of 15 s at 95 °C, 45 s at 58 °C and 45 s at 72 °C. The fluorescent product was detected at the end of the 72 °C extension period. All samples were analyzed by three independent measurements. Primer pairs are listed in Table 1.

### 4.11. Statistical Analysis

Data are presented as the means ± the standard deviation (SD). Statistical significance was analyzed using the Student’s *t*-test or one-way analysis of variance (ANOVA), depending on the normality of the data distribution. *p* < 0.05 was considered as statistically significant. All statistical analyses were performed using Sigma Stat v3.5 (Systat Software Inc., Chicago, IL, USA).

## Figures and Tables

**Table 1:** Primer pairs for qRT-PCR.

Gene	Direction	Primer Sequence
Actin	Sense	GGTTCACTTTTT-CAAGCAGTAGG
	Anti-sense	GTGGTAATCCACTTTCATCCATT
c-Myc	Sense	AAC-TGGAACGGTGAAGGT
	Anti-sense	CCTGTAACAACGCATCTCAT
Cyclin D1	Sense	ACA-TCTTCCAGGAGTACCC
	Anti-sense	CTTGGTGAGGTTTGATCCG
